# Message Effectiveness of Fear Appeals in Vaccination Communication Campaigns: A Systematic Review

**DOI:** 10.3390/vaccines12060653

**Published:** 2024-06-12

**Authors:** Yam B. Limbu, Bruce A. Huhmann

**Affiliations:** 1 Department of Marketing, Montclair State University, Montclair, NJ 07043, USA; 2 Department of Marketing, Virginia Commonwealth University, Richmond, VA 23284, USA; bahuhmann@vcu.edu

**Keywords:** fear appeal, vaccination intentions, vaccine attitudes, message effectiveness, systematic review

## Abstract

This systematic review of 54 cross-disciplinary peer-reviewed causal empirical studies helps public health officials, researchers, and healthcare professionals better comprehend the effects of fear appeals in vaccine promotional campaigns on message processing, persuasion, vaccination attitudes, and vaccination intentions. This review documents inconsistent findings across studies, which it attempts to clarify by considering differences in research designs, sample populations, and outcomes measured. In general, we find that fear appeals increase risk perceptions, message involvement, and vaccination attitudes. However, fear appeals have less influence on vaccination intentions, especially among female and general adult populations or populations from the U.S. and other Western cultures. On the other hand, the effect of fear appeals on vaccination intentions is stronger among student populations and those from China (People’s Republic of China and Hong Kong) and other non-Western cultures. Also, fear appeals are less persuasive when promoting COVID-19 vaccines and boosters than they are for other vaccines (e.g., HPV, influenza, MMR). Future research should compare fear appeal effectiveness in messages across vaccines or when combined with other executional elements, such as the endorser or type of evidence provided. Finally, future studies should explore other methodological approaches and measure underexplored message outcomes, such as vaccine uptake behavior, in more naturalistic settings.

## 1. Introduction

To encourage vaccinations, health communication campaigns, such as posters, social media posts, public service announcements, and pamphlets, often use emotional appeals in addition to rational appeals [1,2]. Rational appeals present facts about the usage, features, attributes, and risks or benefits of a product (e.g., a vaccine), as well as comparison, statistical, or research information. In contrast, emotional appeals attempt to evoke either positive or negative affective responses to influence processing, evaluations, or behavioral responses [3].

Negative emotional appeals appear in many types of health promotions, such as 14% of direct-to-consumer advertisements for prescription medications, 5.1% of advertisements for over-the-counter medications, and 9.1% of dietary supplement advertisements [3]. Among negative emotional appeals, fear appeals tend to be one of the most effective to use in motivating the public to engage in protective behaviors [4]. Fear appeals attempt to motivate receivers to reduce the communicated threat by adopting the recommended change in attitude, intention, or behavior. A closely related concept is loss framing, which attempts to motivate receivers by emphasizing the negative consequences, such as disease severity, resulting from not adopting a recommended behavior (e.g., vaccine uptake), as opposed to gain framing, which emphasizes the positive consequences of adopting the behavior [5,6,7]. Because fear appeals can positively influence attitudes, intentions, and behavior [8], they are widely studied in health communication and promotion contexts [2].

Prior research has investigated fear appeals’ efficacy in improving vaccine message processing and attitudes as well as encouraging vaccination intentions and behaviors. Unfortunately, it is unclear when and how to best apply fear appeals to create effective vaccine-related health promotions or communication campaigns due to inconsistent and contradictory findings, the investigation of different message outcomes with different populations, and the inclusion of different boundary conditions.

However, it is important for public health officials and healthcare professionals to understand how to best use fear appeals to motivate vaccination behavior to protect individuals and populations from disease. The ability to successfully use motivation beyond rational appeals, such as fear appeals, can be helpful because vaccine hesitancy toward specific vaccinations and boosters is strong among several populations [9,10,11].

Thus, a systematic review is needed that provides structure to the prior literature to clarify the effect of fear appeals on message outcomes. Hence, the purpose of our investigation is to systematically identify previous studies of the use of fear appeals in vaccination campaigns to provide a comprehensive understanding of their effectiveness in achieving outcomes related to message processing (e.g., attention, comprehension, and memory) and persuasion (e.g., attitudes, vaccination intentions, and vaccination behavior). To accomplish this, the current review aims to answer several key research questions.

**RQ1.**  *How effective is the use of fear appeals in vaccination campaigns?*

**RQ2.**  *How do fear appeals impact the effectiveness of COVID-19 versus other vaccination types (e.g., HPV, influenza)?*

**RQ3.**  *How does the impact of fear appeals differ across countries and cultures?*

**RQ4.**  *How does the impact of fear appeals vary across populations?*

**RQ5.**  *Under what conditions has the influence of fear appeals on message effectiveness been found to change in strength or direction?*

**RQ6.**  *What intervening factors have been found to mediate the influence of fear appeals on message effectiveness?*

In addition to improving public health officials’ and healthcare professionals’ understanding of the overall efficacy of fear appeals in vaccination campaigns, this review contributes to the literature in additional ways. This study represents the first systematic review of fear appeals’ effect on vaccination communication outcomes. It also demonstrates how fear appeals’ effectiveness varies across vaccine types (e.g., COVID-19, influenza, HPV), populations, and other moderating conditions. In addition, it documents the explanatory power of various mediators that have been explored in prior research, which improves our understanding of the influences on fear appeals’ impact on message processing and persuasion.

## 2. Method

We carried out this systematic review following the guidelines recommended by the Preferred Reporting Items for Systematic Reviews and Meta-Analyses (PRISMA) [12].

### 2.1. Inclusion Criteria

This review included quantitative empirical studies published in peer-reviewed journals and written in English. We included studies that examined the impact of fear appeals on various vaccination-related message outcomes, including information search and processing, word-of-mouth/advocacy, attitudes, and intentions. We excluded qualitative studies, conference proceedings, grey literature, reviews, dissertations, and books. We included only causal studies (e.g., laboratory and field experiments), whereas descriptive studies, such as correlational surveys that simply measured perceptions (e.g., fear of vaccination/disease, severity, susceptibility), were excluded. We also used journal quality as an inclusion criterion; we did not include studies published in journals that are not indexed in one of Scimago’s top three Scientific Journal Ranking tiers (Q1, Q2, or Q3).

### 2.2. Study Identification

We searched PubMed, Web of Science, and Scopus for relevant articles without any time limit until April 2024. PubMed is a commonly used search tool in the health literature. Web of Science and Scopus are the two largest and most commonly used literature databases for searching multidisciplinary literature. We used combinations of these search strings in each database:

1. “fear appeal*”
2. fear OR threat OR scare OR “loss frame” OR “negative frame” OR “shock tactic” OR “risk message” OR “risk perception” OR “risk communication”
3. vaccin* OR immuniz* OR shot* OR inoculat* OR booster.

We generated various variants of search strings to execute exhaustive queries in each database by combining search terms with the Boolean operators.

### 2.3. Search Strategy

**Figure 1:**
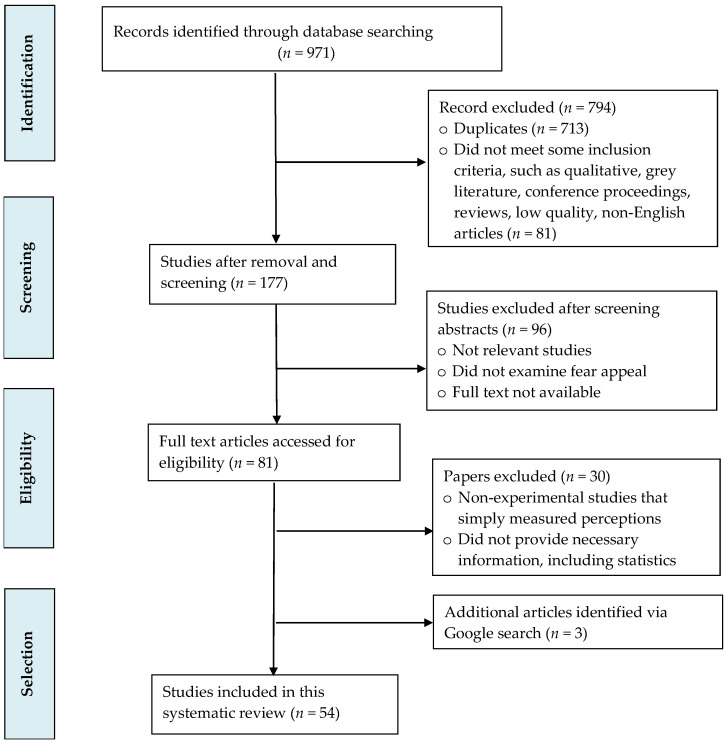
PRISMA flow diagram showing the search strategy and study selection process.

The PRISMA flow diagram (see Figure 1) presents the article selection process. This diagram summarizes the number of records identified, screened, and excluded; the reasons for exclusion; and the number of studies included in this review [12]. We applied no restrictions on publication year or study population.

We retrieved 971 records from electronic databases, which consisted of 388 records from PubMed, 329 from Web of Science, and 254 from Scopus. After removing 713 duplicates and 81 studies that did not meet some inclusion criteria (e.g., qualitative studies, grey literature, conference proceedings, reviews, non-English studies), 177 records were retained. We removed studies that did not meet the inclusion criteria related to journal quality. We verified that the journals that published each study are indexed in one of Scimago’s top three Scientific Journal Ranking tiers (Q1, Q2, or Q3). Next, two authors independently screened all titles and abstracts of the remaining records after deduplication. After screening titles and abstracts, 96 records that did meet the inclusion criteria were eliminated. Finally, the two authors assessed the remaining 81 full-text articles in line with the inclusion and exclusion criteria described above. Any discrepancies between them were resolved by discussion and consensus. Of these, 30 articles did not meet the eligibility criteria. Additionally, we identified three more articles through Google search. Fifty-four studies were included in this systematic review.

### 2.4. Data Extraction

The same two authors independently extracted data from the selected studies. Any disagreements were resolved by consensus. A deductive approach was used, and the key characteristics of the included studies were extracted in line with research questions using a predefined data extraction sheet, which included vaccination types (e.g., COVID-19, HPV, influenza), populations (e.g., general adult population, student), statistically significant and non-significant main effects of fear appeals on attitudes and intentions, mediators, and moderators. We also extracted general information such as the author’s name, publication year, sample size, study design, stimulus, and theory.

## 3. Results

### 3.1. Characteristics of Articles Included in This Review

**Figure 2:**
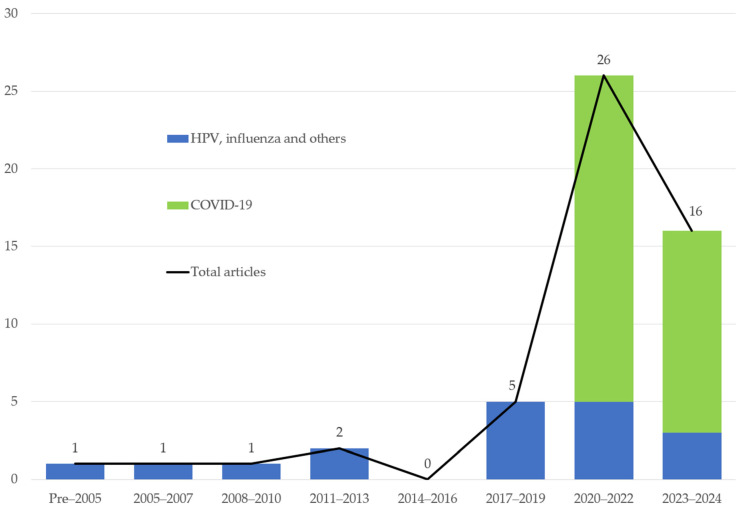
Number of studies of fear appeals in vaccination communications over time.

As presented in Table 1, this study reviewed fifty-four articles from thirteen countries. Twenty-six studies (48.19%) were carried out in the United States, eleven in China/Hong Kong, and four in the United Kingdom. Twenty-six studies (48.19%) were conducted in North America, fifteen in Asia, seven in Europe, two in Africa, and one in Australia. No research was carried out in South America. As shown in Figure 2, most studies were published from 2021 to 2023: seventeen in 2022, fifteen in 2023, and nine in 2021.

Thirty-five studies (64.81%) investigated the COVID-19 vaccine, nine investigated HPV, five investigated influenza, two investigated MMR, and four investigated others. As shown in Figure 2, research on fear appeals in COVID-19 vaccination communications has led to a spike in research activity, but the number of studies on fear appeals in communications regarding other vaccines has been steadily growing for the past few decades.

As shown in Table 1, all studies were cross-sectional. The vast majority of the studies (88.89%, *n* = 48) conducted between-subjects randomized experiments, four conducted within-subjects designs, and two conducted mixed designs. Most studies created a general message without specifying a particular medium. Other frequently used stimuli included social media posts, booklets, leaflets, flyers, posters, and public service announcements.

The vast majority of studies (59.26%, *n* = 32) used a theoretical perspective of framing, followed by the Extended Parallel Process Model (EPPM), Arousal Theory, Health Belief Model, Protection Motivation Theory, and Theory of Planned Behavior. Although a variety of theories have been used to explain the effects of fear appeals in COVID-19 vaccination communications, the EPPM, framing, and Arousal Theory have primarily been used with studies of fear appeals in HPV, influenza, MMR, and other vaccination communications.

Most studies (61.11%, *n* = 33) investigated the general adult population, eleven students, six women, two African Americans, and two parents. The included studies recruited 27,323 respondents, with an average sample size of 514.94 (standard deviation = 1248.21), ranging from 75 to 7064. Whereas several sample populations have been studied with vaccination campaigns for most vaccination types, fear appeals in HPV vaccination campaigns have generally focused on younger and female sample populations.

### 3.2. Overall Main Effect of Fear Appeals across Outcomes

**Figure 3:**
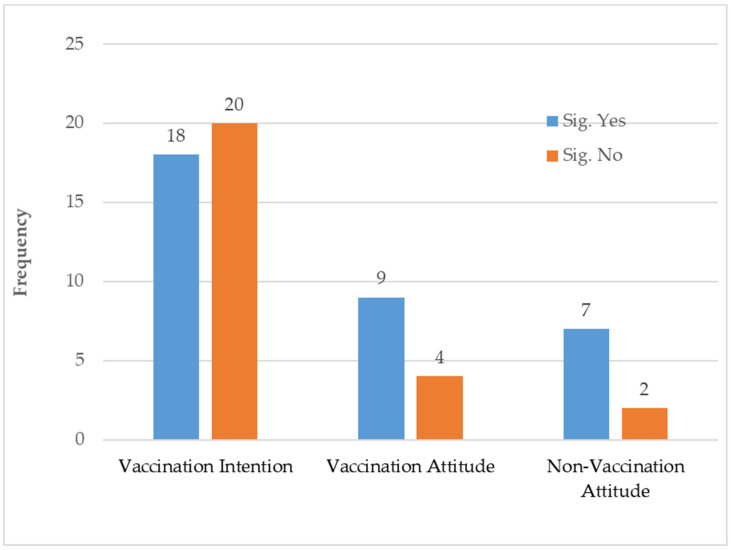
Overall number of statistically significant (*p* < 0.05) versus non-significant main effects of fear appeals on attitudes and intentions.

Thirty-eight studies (70.37%) examined the effectiveness of fear appeals on vaccination intentions (see Figure 3 and Table 2). These studies reported mixed results. Eighteen studies (47.37%) reported statistically significant positive main effects (e.g., [35,37,47,49,57,59]). However, an additional four investigations demonstrated a negligible or a negative/backfire effect of fear messages on vaccination intentions. For example, Brooker [56] revealed a negative impact of fear appeals on influenza vaccination intentions. Hing et al. [22] reported a backfire effect of a negative attribute framing to influence COVID-19 vaccination intentions among Malaysian adults. Similarly, Liu et al. [29] showed that the presence of fear appeals in COVID-19 vaccine campaign posters elicited lower levels of vaccination intentions among Chinese adults than those without fear appeals. Also, the loss frame was less effective in motivating people to vaccinate against COVID-19 than the gain frame among Chinese college students [46].

Thirteen studies (24%) examined the direct effect of fear appeals on vaccination-related attitudes (see Figure 3 and Table 2). Of these, nine studies (69.23%) found statistically significant main effects. Two studies revealed a statistically significant positive influence of a loss-framed message on the perceived outcome efficacy (perceived benefits and costs of receiving an MMR vaccination) [60] and perceived net benefit (the ratio of perceived effectiveness to perceived side effects of a COVID-19 vaccine) [27]. Fear messages were positively associated with attitudes toward vaccination against Ebola [64] and the coronavirus [36]. On the contrary, Brooker et al. [56] found a negative effect of a mild fear message on the perceived need for the vaccine, liking of the vaccine, and attitudes toward the vaccine advertiser. Two studies examined vaccine safety and risk. Loss-framed messages were more effective than gain-framed and emotional–rational messages in reducing the risk perceptions of a COVID-19 vaccine [21]. Another study revealed that people receiving loss-framing information, compared to gain-framing information, considered the COVID-19 vaccine safety risk to be less important in decision-making [41].

Non-vaccination attitudes are attitudes toward objects other than the vaccine in these reviewed studies of fear appeals in vaccination communications, such as the perceived susceptibility and severity of contracting a disease. Seven investigations (77.78%) reported statistically significant direct effects of fear messages on non-vaccination attitudes. However, the findings were mixed. For example, in a study by Vaala et al. [39], fear appeals favorably impacted the perceived threat of COVID-19; however, Wang F. et al. [43] found a negative effect. Furthermore, compared to hope-oriented visual communication, fear-oriented visual communication was less effective at increasing the perception of infectious COVID-19 variants as a health threat [34].

A handful of studies examined outcomes other than attitudes or behavioral intentions. Avery et al. [47] found a positive effect of fear appeals in improving message recall; individuals who reviewed a fear visual in an HPV vaccination campaign flyer scored higher on message recall. On the other hand, fear appeals were negatively related to attitudes toward a COVID-19 vaccination message [31].

### 3.3. Main Effect of Fear Appeals on Vaccination Intentions between COVID-19 and Other Vaccines

As presented in Table 3, twenty-one studies (38.89%) examined the impact of fear appeals on COVID-19 vaccination intentions. Of these, only ten (47.6%) reported statistically significant main effects. On the other hand, two-thirds (66.7%) of the studies that explored the effects of fear appeals on intentions to receive other vaccines (e.g., HPV, influenza, MMR) reported significant results. Although the observed difference between the two groups was not statistically significant (χ^2^ = 1.117, *p* = 0.290), the difference in the proportion of studies reporting statistically significant effects seems to indicate that fear appeals were less effective in motivating people to receive a COVID-19 vaccine than other vaccines.

### 3.4. Main Effect of Fear Appeals on Vaccination Intentions across Countries and Cultures

Table 3 shows that the impact of fear appeals on vaccination intentions significantly differed across countries and cultures. Fear appeals were more effective in promoting vaccination intentions among the Chinese than Americans (87.5% vs. 33.3%, χ^2^ = 6.135, *p* = 0.013). Similarly, fear messages were associated with increased vaccination intentions in non-Western countries compared to those in Western countries (81.8% vs. 35%, χ^2^ = 6.229, *p* = 0.012).

### 3.5. Main Effect of Fear Appeals on Vaccination Intentions across Populations

The reviewed studies investigated various populations, including the general adult population, students, women, and parents. The effectiveness of fear appeals on vaccination intentions differs across these populations. Table 3 shows that fear appeals in vaccination messages were more effective in motivating students (66.7%) than the female (40%) or general adult populations (52.2%). However, the observed differences between the three groups were not statistically significant due to the small number of studies investigating certain populations (χ^2^ = 0.795, *p* = 0.672).

### 3.6. Moderatoring Effects

Twenty-two studies (38.89%) found moderating effects of various factors on the relationship between fear appeals and vaccination intentions. Five studies (9.26%) reported statistically significant interaction effects between fear appeals and perceived response efficacy [45,50,58] or perceived benefits [16,25]. Nan et al. [58] found that a loss-framed message was more effective than a gain-framed message at inducing vaccination intentions when perceived vaccine efficacy was low. Three studies examined the moderating effects of familiarity and prior experience. They reported significant interaction effects between fear appeals and vaccine familiarity [14], vaccine side-effect familiarity [13], or a prior experience of vaccination [60]. Two studies demonstrated the moderating effect of self-efficacy towards vaccine immunization on the relationship between fear appeals and vaccination intentions [25,50]. Trust-related variables, such as trust in family physicians [26] and trust in vaccine benefits [32], also moderated the impact of fear appeals on vaccination intentions.

Additionally, anti-vaccine attitudes moderated the effect of fear messages on COVID-19 vaccination intentions. Two studies reported a significant interaction effect between fear appeals and the perceived threat of disease [25] or perceived vaccine infection risk [42]; the effect of loss-framing information on COVID-19 vaccine acceptance was stronger among those with a higher perceived infection risk and among unvaccinated people with lower confidence in vaccine safety. Other moderators that influenced the impact of fear appeals on vaccination intentions included psychological uncertainty [24], avoidance motivation [51], social norms (individual vs. group) [29], and visual attention [47].

### 3.7. Mediating Effects

Eleven studies (20.37%) reported statistically significant intervening factors mediating the relationship between fear appeals and vaccination intentions. Three studies established the mediating role of the perceived benefit of a recommended vaccination; these studies showed that perceived outcome efficacy [60] and perceived net benefits [28] mediated the impact of loss-framed messages on vaccination willingness. In a study by Carcioppolo et al. [48], perceived response efficacy (i.e., perceived effectiveness of a recommended HPV vaccine) mediated the relationship between fear messages and vaccination intentions; women who viewed a loss-framed message (genital warts message condition) were more likely to perceive the vaccine as efficacious, which in turn positively influenced their vaccination intentions.

In a study by Gursoy et al. [21], perceived vaccination risk mediated the relationship between fear messages and vaccination intentions; loss-framed messages reduced perceived vaccine risk, which in turn increased vaccination intentions. Likewise, Barnes et al. [14] demonstrated that worry regarding or the severity of vaccine side effects could mediate the impact of fear messages on vaccination intentions; however, their results showed that negatively framed messages were inferior to positively framed messages.

Hong and Hashimoto [23] examined the intervening role of message elaboration. They found that fear messages that showed potential negative consequences from one’s failure to receive a COVID-19 vaccination led to greater message elaboration, which enhanced intentions toward vaccination. Luo et al. [54] revealed hope and cognitive elaboration as serial mediators in the impact of fear appeals on HPV vaccination intentions; however, compared to the gain-framed message, the loss-framed message reduced hope, which in turn decreased cognitive elaboration, lowering HPV vaccination intentions.

Two studies examined the mediating role of vaccination attitudes in the relationship between fear messages and vaccination intentions [23,36]. Their results showed that loss-framed appeals led to more favorable attitudes toward vaccination than gain-framed appeals and sequentially predicted increased vaccination intentions. Zhang et al. [46] found that, compared with the gain framing, the loss-framed intervention was more effective in stimulating Chinese college students’ perceived behavioral control (their assessments of the difficulty of completing the COVID-19 vaccine uptake), which in turn positively related to their willingness to receive vaccinations.

## 4. Discussion

### 4.1. Main Findings

This systematic review sought to understand the current landscape of empirical research on the effectiveness of fear appeals in vaccination campaigns regarding message outcomes. Using rigorous systematic review methods, 54 articles published to date met the inclusion criteria, including 42 published in 2020–2024. Thus, interest in this topic among researchers has been increasing greatly over the past few years.

In general, fear appeals favorably affected many message outcomes, including information search and processing, word-of-mouth/advocacy, attitudes, and intentions.

Only one study measured behavior. However, Sato and Takasaki [6] found no difference in tetanus vaccine uptake following exposure to a flipchart containing image and text information with or without a fear appeal.

It is unsurprising that fear appeals had a greater effect on upstream message outcomes, such as information processing and attitudes, than downstream message outcomes, such as vaccination intentions or vaccine uptake behavior. Studies of message outcomes in response to emotional appeals in advertising show stronger effects on brand cognitions and attitudes than purchase intentions, according to Brown et al.’s [65] meta-analysis. In general, changes in attitudes lead to less change in terms of intentions and behavior. Kim and Hunter’s [66] meta-analysis found a stronger correlation between attitudes and behavioral intentions (*r* = 0.64) than between attitudes and behavior (*r* = 0.47) or intentions and behavior (*r* = 0.46).

Fear appeals also exhibited negative effects in some studies. Fear appeals reduced communication quality perceptions [29], including perceptions of social media message effectiveness and shareability [20]; the perceived threat of disease [43]; vaccination risk perceptions [21], as well as the perceived need for and safety of the vaccine [41,56]; vaccine attitudes [56]; response efficacy [39]; perceived social pressure to be vaccinated [43]; and vaccine intentions [22,29,46,56].

### 4.2. Population

Prior research has investigated the response of several sample populations to fear appeals in vaccination communication campaigns. The effectiveness of fear appeals regarding vaccination intentions differs across these populations, such that fear appeals in vaccine communications have a somewhat larger influence among students than general adult or female populations. One possible explanation is that fear appeals may become less effective at motivating responses to vaccine communications with repeated exposure; so, somewhat older populations who have encountered more fear appeals in vaccine communications over their lives are less persuaded by them. Such “wearout” effects of fear appeals have been studied in other contexts, such as anti-speeding messages intended to promote driving safety [67]. Another possible explanation is that student populations contain fewer people who are opposed to vaccines than other populations, as many schools require all attendees to have a mandated set of vaccinations prior to enrolling. Thus, students, because they have agreed to receive other vaccinations in the past, may be more easily persuaded by vaccine communications in general.

The reviewed studies show that the use of fear appeals to promote vaccinations has also been investigated with populations from many different countries. The effect of fear appeals in vaccine communication campaigns has most commonly been studied using American, Chinese (People’s Republic of China and Hong Kong), and English samples. A few studies have also been conducted on samples from other parts of Asia, Europe, and Africa.

In a vaccine communication context, the current review found that message outcomes, especially vaccination intentions, tended to be less likely to change when studies exposed people from Western rather than non-Western cultures to fear appeals. Similarly, fear appeals were less likely to influence message outcomes in studies with American as opposed to Chinese sample populations.

One possible explanation for these results is the cultural orientation of collectivism versus individualism [68]. East Asian cultures tend to value collectivism, which emphasizes serving the interests of and sharing among the members of a larger group. On the other hand, Western cultures value individualism, which emphasizes viewing the self as distinct from others, individual freedom and power, and personal success. Prior research in different contexts has found that the effectiveness of fear appeals can differ between collectivist and individualist cultures. One study conducted in China and the U.S. found that collectivists were less likely than individualists to perform data backups to protect their personal data following a fear message regarding information security [69]. A study examining attitudes toward condom use to prevent AIDS found that fear appeals were more effective with individualist students than with collectivist students [70]. However, another study found that risk perceptions and message acceptance were more sensitive to the level of fear appeal in an anti-smoking advertisement for respondents from a collectivist culture (South Korea) than they were for those from an individualist culture (the U.S.) [71]. Due to such conflicting results across contexts, a meta-analysis of fear appeals in psychological research found them to be equally effective in individualist and collectivist cultures [8]. Although the pattern of the effectiveness of fear appeals regarding vaccination intentions is complicated across the reviewed studies, a difference is discernable in the reviewed studies between individualist and collectivist cultures. Most studies conducted in an individualist culture show that fear appeals often had no effect on Americans’ vaccination intentions [15,16,17,18,23,24,30,33,36,38,45,48,51,53,58,59], although a few found that fear appeals occasionally improve [10,49,62] or harm Americans’ vaccination intentions [56]. In contrast, studies conducted in a collectivist culture show that fear appeals primarily increase Chinese vaccination intentions [19,27,28,42,44,50,57], although occasionally, fear appeals decrease Chinese vaccination intentions [29,41,46]. However, it is rare that fear appeals have no effect on Chinese vaccination intentions [54].

### 4.3. Explanatory Variables and Boundary Conditions

In terms of promoting health-related behaviors and products, mediator and moderator variables are important ways that researchers can learn more about the effectiveness of a particular message execution or communication technique and improve predictions of why and when to expect it to influence message outcomes. This systematic review identified many explanatory variables through which fear appeals in vaccination campaigns have been found to influence message outcomes. Some researchers have investigated mediators related to the vaccine’s risks, benefits, or efficacy [14,21,23,48,60]. Others have investigated mediators related to message reactions [24], perceived behavioral control [46], and vaccine attitudes [23,36].

This systematic review identified many individual facilitators and inhibitors of audience responses to fear appeals in vaccination campaigns that have been studied so far. Some researchers have investigated boundary conditions related to other message executions in vaccine communications in addition to fear appeals included in the vaccine communication, such as controlling language and disgust appeals [31]; other-referencing messages, which refer to risks to and the safety of other people [23]; a cash incentive offered for vaccination [6]; and message source types [57]. Other moderators related to risks, benefits, and efficacy [16,23,25,39,42,45,50,58]. Some moderators relate to an individual’s past behavior [42,51,60], baseline intentions [14], self-efficacy [25,39], trust [26,32,39], familiarity with the vaccine or its side effects [13,14], anti-vax attitudes [55], psychological uncertainty [24], avoidance motivation [51], and issue relevance [61]. Finally, other moderators investigated so far include visual attention [47]), social media likes [50], and social norms [29].

### 4.4. Theoretical Explanations for Fear Appeal Efficacy

The current review of the prior literature finds that several theories relating to the effectiveness of fear appeals have been investigated in relation to vaccine communication campaigns. One well-supported theory specifically developed to explain how fear appeals impact health communication outcomes is Roger’s Protection Motivation Theory (PMT) [4,72]. It suggests that people look for the best match between how they appraise threats and possible coping strategies. It also considers intrinsic versus extrinsic rewards, how severe the threat is perceived to be, how vulnerable someone feels, the perceived effectiveness of each possible response, self-efficacy, and the costs to lessen the threat. Following this process, people will choose to either take a protective action (e.g., a vaccination) or not.

The Extended Parallel Process Model (EPPM) extends the PMT [7,73]. EPPM suggests that fear appeals encourage message recipients to believe that they are susceptible to severe consequences from the threat. The current review found that almost three times as many reviewed studies relied on the EPPM than the PMT as a theoretical foundation.

The most common theoretical foundation appearing in the reviewed studies involved message framing, either positive versus negative attribute framing or framing outcomes as losses rather than gains. Framing arises from Kahneman and Tversky’s [74] Prospect Theory, which holds that how a message is framed affects recipients’ responses. For example, positive framing might state the number or proportion of those protected from disease by a vaccine or unaffected by a side effect, whereas negative framing might state the number or proportion who suffered a disease or side effect. Gain-framing typically discusses the benefits of the suggested action. An example gain-framed message might state, “if you receive this vaccine, you will stay healthy.” In contrast, loss-framing discusses the costs of not complying. An example loss-framed message might create a fear appeal by stating “if you are unvaccinated, you will suffer negative consequences, such as illness or death.” Prior research also discussed framing in terms of cognitive appraisal theory, which suggests that how people interpret adverse situations or high uncertainty can elicit fear [30].

A few studies adopted a theoretical approach focused on arousal. Fear arousal theory holds that fear appeals increase arousal, which increases both message processing and defensive responses, such as message avoidance [5]. One study in the current review found that pro-vaccination social media messages containing fear appeals also aroused defensive responses, such as reduced sharing and the hardening of stances against vaccination [20]. Whether a message’s emotional appeal is positive or negative also matters. A recent review of advertising appeals in health communication found that positive emotional appeals are generally more effective than negative emotional appeals or appeals that try to elicit both positive and negative emotional responses [2]. However, negative emotional appeals have a greater effect on attitude change than positive emotional appeals [75], because negative emotional appeals (e.g., fear appeals) create anxiety that the message suggests can be reduced by adopting the message’s recommendations [3].

The Health Belief Model [76] and the Theory of Planned Behavior [77] appeared about equally as often as arousal as a theoretical foundation in the reviewed studies. The Risk Perception Attitude Framework and Terror Management Theory each appeared once [33,49]. Finally, two studies used reactance to predict message outcomes in response to fear appeals in vaccine communications [31,39].

### 4.5. Limitations and Directions for Future Research

One limitation of this review is its focus on reviewing findings from causal studies only. Other reviews may want to examine studies that used other methods, such as qualitative research, to obtain a fuller picture of the state of research on the effectiveness of including fear appeals in messages to encourage vaccination.

Also, the search strategy focused on peer-reviewed causal studies in the PubMed, Web of Science, and Scopus databases. However, given the type of review and paucity of studies eligible for inclusion, no formal quality assessment was conducted. Once more causal studies have been conducted, a meta-analysis of fear appeals in vaccination communications could include a formal quality appraisal to help develop a sensitivity analysis for testing whether the quality of studies on this topic systematically biases the effect sizes. However, future researchers may want to consider whether this is worthwhile, given that appraisals of study quality are complex and potentially subjective [78,79,80]. Further, recent research has shown that the removal of bias threats using quality appraisals has a negligible effect on results [81].

In terms of providing a way forward in investigations of this topic, this systematic review’s literature analysis identifies several gaps. These gaps provide several potential avenues for future research.

First, this review uncovered differences in the effectiveness of fear appeals in vaccine communication campaigns across vaccine types. Fear appeals motivated message outcomes less when encouraging COVID-19 vaccinations as opposed to other types of vaccinations. Fear appeals were effective, with less than half of the message outcomes measured in prior research, when used in communications regarding COVID-19 vaccinations, compared with almost three-fourths with other vaccinations, including Ebola, H1N1, HPV, Influenza, Meningitis, MMR, and Tetanus. However, it is difficult to ascertain from prior research whether this is due to the inappropriateness of using fear appeals in the context of COVID-19 vaccinations or the particular fear appeals chosen or whether any message execution would be less effective in COVID-19 vaccine communications given the polarization of political and cultural messages regarding the COVID-19 vaccines and the distrust in medical science and vaccines among segments of the population engendered by these polarizing political and cultural messages (e.g., [15]). Future research could investigate these potential explanations further by examining mediators of relationships for a non-fear versus fear appeal on another vaccine’s uptake or intentions versus a COVID-19 vaccine or booster’s uptake or intentions. Alternately, future studies could compare the message outcomes of a COVID-19 vaccine versus another vaccine before and after exposure to a communication campaign with or without a fear appeal. Future research could also directly compare the effectiveness of a fear appeal in vaccination communication campaigns for COVID-19 against a control condition of another disease that the sample population views as similarly severe or intense.

Second, the studies in this review almost exclusively posited a linear relationship between fear appeal strength and effectiveness in promoting vaccine message outcomes. However, fear may actually exhibit a non-monotonic inverted-U shaped relationship such that fear appeals are more effective in promoting message outcomes at a medium level than when weaker or stronger. This might help explain the inconsistent findings in the reviewed studies where fear appeals in vaccination communications often improve but, at times, worsen message outcomes. Although they examined only a low versus a high fear appeal and did not posit an inverted-U relationship, Keller and Block [5] found that low fear appeals did not elicit sufficient elaboration regarding the problem or threat, which lowered attitude favorableness, but high fear appeals needed to reduce elaboration on the problem or threat to be effective. Studies of tobacco warnings found that the inverted-U relationship was easier to observe when studies controlled within-individual differences in response [82]. In the reviewed studies, only Dillard and Shen [62] investigated a curvilinear relationship. They examined and confirmed that the level of fear over time had an inverted-U shape that was predictive of the intention and behavior of checking one’s meningitis vaccination status. In Tannenbaum et al.’s [8] meta-analysis of studies in psychology, the test of a linear versus a curvilinear relationship between fear appeals and attitudes, intentions, or behavior was inconclusive. However, Sapolsky [83] posits that neurobiological endpoints should exhibit an inverted-U relationship with stress such that these endpoints rise from under-stimulation to a peak and then decline as overstimulation intensifies. Similarly, Boywitt [84] finds an inverted-U relationship with memory for emotionally arousing pictures. Thus, future research in vaccine communication should test the effectiveness of fear appeals of low, medium, and high intensities to determine if fear appeal strength exhibits an inverted-U function in regard to message outcomes, such as vaccination intentions. Knowing the most effective level of fear appeal in this context would be of great help to those designing vaccination communications.

In the current review, we sought causal studies that applied various methodological approaches; however, the existing literature was mostly limited to experimental studies conducted using online lab methods (e.g., using Qualtrics to expose respondents to a message stimulus with or without a fear appeal, followed by items used to measure their self-reported responses). Several laboratory and online lab experiments included simple messages, including a fear appeal manipulation within vignettes or scenarios. Others investigated message outcomes associated with a fear appeal manipulation embedded within a specific type of communication, such as social media posts, public service announcements, advertisements, posters, and booklets. Experiments are useful for manipulating and testing message executions, such as fear appeals, but future research might consider other methods. For example, a field study could be designed to measure behavioral responses in an environment that includes messages with or without fear appeals. Only two of the reviewed studies [6,40] employed field studies. In addition, before-and-after studies, time-series analysis, or causal machine learning could provide interesting tests of fear appeals’ effects in vaccine communications regarding message outcomes.

The current review found that the efficacy of fear appeals was examined for a variety of message outcomes. The most commonly investigated outcome was vaccine intentions, followed by attitudes. Future research could explore some of the previously uninvestigated or understudied message outcomes, such as fear appeals’ effect on attention to and comprehension of vaccine communications as well as vaccine uptake and overcoming vaccine hesitancy. It would also be interesting to see future research use search, vaccine enrollment, word-of-mouth, or click-through data from search engines, social media sites, or websites to investigate the effects of exposure to fear appeals in online vaccine communications (e.g., banner ads and social media posts) on actual behavior in a natural online setting.

Vaccine intentions were often measured in the review studies, but only one study measured vaccine uptake [6]. Despite the importance of vaccination intentions as a message outcome, prior research has found that intentions are a less-than-perfect surrogate for actual behavior. According to some meta-analyses, intentions only account for 28% of the variance in behavior. In addition, a moderate-to-large effect on intentions only results in a small-to-moderate effect on behavior [85,86].

The relationship between intentions and behavior can be strengthened or weakened by social influence, unexpected situational factors or the environmental context (e.g., crowding or long queues, time pressure, stock-outs, absenteeism of crucial personnel, the weather, or unpleasant settings), the receipt of new information or persuasion communications, changes in price or other costs, one’s perceived degree of control (e.g., government mandates may lead to vaccine uptake despite one’s intention to remain unvaccinated or, alternatively, may reduce vaccine uptake in individualist cultures due to reactance), changes to one’s circumstances (e.g., the cancellation of an international trip may stop one from receiving an intended vaccination), and the passage of time between intention declaration and behavior performance. While some of these factors and the intention–behavior relationship have been explored in other contexts [85,86,87,88], this would be a fruitful area for future research on vaccine communication. For example, if a fear appeal can increase vaccine intentions, how does the passage of time between exposure to the fear appeal and a potential vaccination appointment affect vaccine uptake? One might posit that the motivating strength of the emotional response to a fear appeal would weaken over time. Similarly, factors (e.g., out-of-stock vaccines or the temporary absence of personnel capable of administering a vaccine) that delay vaccine uptake behavior would also be predicted to weaken its relationship with vaccine intentions following exposure to a fear appeal.

Finally, a minority of studies included in this review revealed significant main effects of fear appeals on vaccination intentions. To improve effectiveness, future research should consider examining the joint effects of fear appeals and other message strategies, such as the message source (e.g., expert, celebrity), appeal (e.g., humor, guilt), framing (e.g., goal, attribute), and evidence type (e.g., anecdotal, statistical), on recipients’ vaccination attitudes, intentions, and behaviors [89].

## 5. Conclusions

Vaccine communication campaigns frequently include fear appeals to motivate the public to protect their health and slow the spread of disease by engaging in preventative behaviors. These fear appeals typically describe a threat related to the possible risks or dire consequences of not receiving a vaccination. However, prior research was somewhat contradictory regarding the effectiveness of fear appeals in vaccine communications. The current review helps clarify the findings from prior studies by examining the effectiveness of fear appeals regarding a variety of message outcomes across different sample populations, types of communications, and diseases as well as the explanatory role of various moderators and mediators. It also identifies gaps that future research should address, including the need for causal studies beyond lab or online lab experiments that can investigate outcomes in more natural message exposure settings, more studies of actual vaccine uptake not just intentions, and comparisons of how effectiveness differs across vaccines or when combined with other executional elements (e.g., other emotional appeals, endorsers, types of evidence). Thus, the current review contributes to the ability of medical and public health professionals to promote vaccination and to researchers’ understanding of the efficacy of fear appeals in motivating attitudinal and behavioral responses related to vaccination communications.

## Figures and Tables

**Table 1:** Characteristics of studies included in this review.

Vaccination Type							
Author(s)	Year of Publication	Country	Population	Sample Size (*n*)	Design	Stimulus	Theory
COVID-19							
Barnes & Colagiuri [13]	2022	UK	Adult	1222	Between-Ss, stratified random, online lab	Leaflet	Framing
Barnes et al. [14]	2023	Australia	Adult	1204	Between-Ss, stratified random, online lab	Infographic	Framing
Borah [15]	2023	USA	Adult	387	Between-Ss, randomized, online lab	CDC Facebook post	Framing
Borah et al. [16]	2021	USA	Adult	387	Between-Ss, randomized, online lab	CDC Facebook post	Framing
Callow & Callow [17]	2021	USA	Adult	583	Between-Ss, randomized, online lab	Government announcement	Framing, TPB
Chen et al. [18]	2022	USA	Adult	539	Between-Ss, randomized, online lab	News article	Framing
Gong et al. [19]	2022	China	Adult	1316	Between-Ss, randomized, laboratory	Message	Framing
Grabe et al. [20]	2023	USA	Black	710	Within-Ss, online lab	Twitter advertising post	Arousal, Valence
Gursoy et al. [21]	2022	USA	Adult	*n*1 = 1020 *n*2 = 266	Between-Ss, randomized, online lab	Message	Framing, PMT
Hing et al. [22]	2022	Malaysia	Adult	5784	Between-Ss, randomized, online lab	Website message	Framing
Hong & Hashimoto [23]	2023	USA	Student	213	Between-Ss, randomized, laboratory	Message	Framing
Huang & Lui [24]	2022	USA	Adult	382	Between-Ss, randomized, online lab	Twitter post	Framing
Jin et al. [25]	2021	Pakistan	Adult	320	Between-Ss, randomized, online lab	Public service announcement	HBM
Juanchich et al. [26]	2023	UK	Adult	*n*1 = 191 *n*2 = 453 *n*3 = 451 *n*4 = 464	Between-Ss, randomized, online lab	Message	Framing
Li et al. [27]	2023a		Adult	1316	Between-Ss, randomized, online lab	Message	Framing
Li et al. [28]	2023b	China	Adult	981	Between-Ss, randomized, online lab	Message	Framing
Liu et al. [29]	2022	China	Adult	859	Within-Ss, online lab	Poster	EPPM
Lu et al. [30]	2023	USA	Student	337	Between-Ss, randomized, laboratory	Advertisement	Framing
Ma & Miller [31]	2022		Adult	564	Mixed design, randomized, online lab	Message	Reactance, EPPM
Masiero et al. [32]	2022	Italy	Adult	634	Between-Ss, randomized, online lab	Message	Framing
Motta et al. [33]	2021	USA	Adult	7064	Between-Ss, randomized, online lab	Newspaper opinion article	Terror Management Theory
Petersen et al. [34]	2022	USA		3022	Between-Ss, randomized, online lab	Graph of the hospitalized for a COVID-19 variant	
Prakash et al. [35]	2022	India	Young adult	228	Between-Ss, randomized, online lab	Public health ministry announcement	Framing, TPB
Reinhardt & Rossmann [36]	2021	USA	Adult	281	Between-Ss, randomized, laboratory	Message	Framing
Sasaki et al. [37]	2022	Japan	Adult	1595	Between-Ss, randomized, online lab	Message	Framing
Taber et al. [38]	2023	USA	Adult	*n* = 589 *n* = 274	Between-Ss, randomized, online lab	Message	Framing
Vaala et al. [39]	2022	USA	Adult	442	Between-Ss, randomized, online lab	CDC Twitter post	Reactance, EPPM
Vepachedu et al. [40]	2024	Ghana	Adult	1494	Between-Ss, randomized, telephone field study	Telephone call	
Wang et al. [41]	2022	Hong Kong	Parent	298	Between-Ss, stratified random, online lab		Framing
Wang et al. [42]	2023	Hong Kong	Adult	1000	Between-Ss, randomized, online lab	Message	Framing, HBM
Wang F. et al. [43]	2022	China	Parent	165	Between-Ss, randomized, online lab	Media reports	EPPM
Ye et al. [44]	2021	China	Student	298	Between-Ss, randomized, online lab	Newsletter	Framing, HBM
Zhang & Marvel [45]	2022	USA	Black	547	Between-Ss, randomized, online lab	Health risk article	PMT, EPPM
Zhang et al. [46]	2023	China	College student	228	Between-Ss, randomized, online lab	Social media story	Framing, TPB
HPV							
Avery et al. [47]	2018		Student	75	Between-Ss, randomized, eye tracking	Flyer	EPPM
Carcioppolo et al. [48]	2013	USA	Female student	442	Between-Ss, randomized, laboratory	Message	Framing, EPPM
Carcioppolo et al. [49]	2017	USA	Male and female of vaccination age	407	Within-Ss, online lab	Advertisement	Risk Perception, Attitude Framework, PMT, EPPM
Chen et al. [50]	2021a	China	Female	478	Between-Ss, randomized, laboratory	Weibo post	EPPM
Gerend & Shepherd [51]	2007	USA	Female student	121	Between-Ss, randomized, laboratory	Booklet	Framing
Kim et al. [52]	2020	USA	Student	269	Between-Ss, randomized, online lab	Message	PMT, EPPM
Kim et al. [53]	2022	USA	Young adult	347	Between-Ss, randomized, online lab	Message	Framing
Luo et al. [54]	2024	China	Female student	175	Between-Ss, randomized, online lab	WeChat post	Framing
Reno & Dempsey [10]	2023	USA	Parent Young adult	*n* = 291 *n* = 409	Between-Ss, randomized, online lab	Message	EPPM
Influenza							
Bender et al. [55] *	2023	Germany	Adult	*n*1 = 332 *n*2 = 320	Between-Ss, randomized, online lab	Leaflet, video	Framing
Brooker [56]	1981	USA	Adult	240	Between-Ss, randomized, laboratory	Booklet	Arousal
Chen et al. [57]	2021b	China	Student	534	Between-Ss, randomized, online lab	Weibo post	EPPM
Nan et al. [58]	2012	USA	Older adult	88	Between-Ss, randomized, laboratory	Health pamphlet	Framing, TPB
Roberto et al. [59]	2019	USA	Student	*n* = 482 *n* = 277	Between-Ss, randomized, laboratory	Message	EPPM
MMR							
Abhyankar et al. [60]	2008	UK	Female	140	Between-Ss, randomized, laboratory	Message	Framing, PMT, TPB
Lu & Yuan [61]	2023	USA	Adult	386	Between-Ss, randomized, online lab	YouTube video blog post	Arousal
Others							
Dillard & Shen [62]	2018	USA	Student	290	Within-Ss, randomized, laboratory	Website message	Arousal
Ecker et al. [63]	2023	UK	Adult	380	Mixed design, online lab	Booklet	Arousal
Ort & Fahr [64]	2018	Europe	Student	447	Between-Ss, randomized, laboratory	Public health website	EPPM
Sato and Takasaki [6]	2021	Nigeria	Female	1660	Between-Ss, stratified random, field study	Image and text description	Framing, HBM

Note: EPPM = Extended Parallel Process Model, HBM = Health Belief Model, PMT = Protection Motivation Theory, TPB = Theory of Planned Behavior, Between-Ss = Between-Subjects Design, Within-Ss = Within-Subjects Design, * Influenza and COVID-19.

**Table 2:** Fear appeal main effects on outcomes and mediator or moderator relationships.

Vaccination Type						
	Year of	Main Effect				
Author(s)	Publication	Predictor	Outcome	Significance	Mediator	Moderator
COVID-19						
Barnes & Colagiuri [13]	2022	Fear	VI	No		Gain x vaccine side effect familiarity → VI
Barnes et al. [14]	2023	Loss	VI (control, <gain)	Yes	Gain → side effect worry/severity → VI	Framing (>control) x unfamiliar vaccine → VI; framing x familiarity x baseline intention → VI
Borah [15]	2023	Loss	VI	No		
Borah et al. [16]	2021	Loss	VI, attitude toward COVID-19 vaccine	No		Loss x perceived benefits → VI, attitude toward COVID-19 vaccine
Callow & Callow [17]	2021	Loss	VI	No		
Chen et al. [18]	2022	Loss	VI, attitude	No		
Gong et al. [19]	2022	Loss	VI	Yes		
Grabe et al. [20]	2023	Fear	Contagion potential of personalized COVID-19 vaccine messages (−)	Yes		
Gursoy et al. [21]	2022	Loss	Perceived vaccination risk (−)	Yes	Loss → perceived vaccination risk → VI	
Hing et al. [22]	2022	Loss	VI (−)	Yes (<)		
Hong & Hashimoto [23]	2023	Loss	VI	No	Loss x low perceived risk of COVID-19 x other referencing message → message elaboration → VI Loss x low perceived risk of COVID-19 x other referencing message → attitude toward vaccine → VI	Loss x other-referencing message → message elaboration Loss x other-referencing message x low perceived risk → attitude toward vaccine
Huang & Lui [24]	2022	Loss	Perceived threat to freedom, anger Vaccine-related beliefs, VI	Yes No	Multiple mediators	Loss x psychological uncertainty (high) → VI, vaccine-related beliefs
Jin et al. [25]	2021	Fear	VI	NR		Fear x perceived threat of COVID-19, perceived benefits of COVID-19 vaccines, self-efficacy → VI
Juanchich et al. [26]	2023	Loss	VI	NR		Loss > Gain among unvaccinated x Trust in family physicians (high) → VI
Li et al. [27]	2023a	Loss	Perceived net benefit, VI	Yes	Loss → perceived net benefit → VI	
Li et al. [28]	2023b	Loss	VI	Yes		
Liu et al. [29]	2022	Fear	VI (−), perceived communication quality (−)	Yes (<)		Fear x social norm (individual vs. group) → VI, perceived information quality
Lu et al. [30]	2023	Fear	VI Hope about the vaccine	No Yes		
Ma & Miller [31]	2022	Fear	VI Attitude toward the message (−), freedom threat, anger, negative cognitions, source derogation	No Yes		Fear (low) x disgust (low) x controlling language (low) → source derogation (−), attitude (+)
Masiero et al. [32]	2022	Loss	VI	No		Loss > gain x trust in vaccine benefit → VI (−)
Motta et al. [33]	2021	Messages emphasizing the personal health risks and collective health consequences of not vaccinating	VI	Yes		
Petersen et al. [34]	2022	Fear vs. hope	Perceived health threat (−), adhere to guidelines (−), safely get through (−), strong measures required (−)	Yes		
Prakash et al. [35]	2022	Loss	VI	Yes		
Reinhardt & Rossmann [36]	2021	Loss	Attitude toward vaccination VI	Yes No	Loss → Attitude → VI (younger adults)	
Sasaki et al. [37]	2022	Loss vs. control group	VI	Yes		
Taber et al. [38]	2023	Loss	VI	No		
Vaala et al. [39]	2022	Fear	Perceived threat of COVID-19, self-efficacy, response efficacy (−)	Yes		Fear → self-efficacy (−), response efficacy (−) x trust (low)
Vepachedu et al. [40]	2024	Fear	VI	No		
Wang et al. [41]	2022	Loss	VI for children, vaccine safety (−)	Yes		
Wang et al. [42]	2023	Loss	VI	Yes		Perceived infection risk (high), severity of condition (high), unvaccinated people with a lower confidence in vaccine safety
Wang F. et al. [43]	2022	Fear	Recommend others to get the COVID-19 vaccine, perceived threat of COVID-19 (−), social pressure to receive COVID-19 vaccine (−)	Yes		
Ye et al. [44]	2021	Loss	VI	Yes		
Zhang & Marvel [45]	2022	Fear	VI	No		Perceived vaccine efficacy (+) → VI
Zhang et al. [46]	2023	Loss	VI (−), PBC	Yes (<)	Frame → PBC → VI	
HPV						
Avery et al. [47]	2018	Fear	VI, message recall	Yes		Fear x visual attention → VI
Carcioppolo et al. [48]	2013	Threat, Message framing (severity)	VI	No	Low Threat → Low Fear → Low VI; Message framing (severity) → Response efficacy → VI	
Carcioppolo et al. [49]	2017	Fear	VI	Yes		
Chen et al. [50]	2021a	Threat	VI	Yes		Threat x efficacy x likes
Gerend & Shepherd [51]	2007	Loss	VI	No		Loss x who had multiple sexual partners, infrequently used condoms, and are high in avoidance motivation → VI
Kim et al. [52]	2020	Fear	Motivation to process HPV protection-related information	Yes		
Kim et al. [53]	2022	Loss	Anticipated regret of not taking HPV vaccine Attitude toward HPV vaccination, VI	Yes No		
Luo et al. [54]	2024	Loss	VI	No	Gain → hope → cognitive elaboration → VI	
Reno & Dempsey [10]	2023	Fear	VI, HPV vaccination information seeking	Yes		
Influenza						
Bender et al. [55] *	2023	Side effect message (gain-framed (vs. standard))	VI, adverse event expectation for COVID-19 (−), cost–benefit ratio for influenza (−)	No		Gain-framed side effect x anti-vaccine attitudes → VI (−) for COVID-19
Brooker [56]	1981	Mild fear (vs. straight-forward)	VI (−), perceived need for the vaccine (−), liking for the vaccine (−), attitude toward the advertiser (−)	Yes		
Chen et al. [57]	2021b	Fear (visible source)	VI, flu-related information seeking	Yes		Visible source x receiver source x technological source → flu-related information seeking
Nan et al. [58]	2012	Loss	VI	No		Loss > gain x vaccine efficacy (low) → VI
Roberto et al. [59]	2019	Threat	Perceived severity, susceptibility, fearVI	Yes No		
MMR						
Abhyankar et al. [60]	2008	Loss	VI, outcome efficacy	Yes	Frame → outcome efficacy → VI	Loss x past MMR decision → VI
Lu & Yuan [61]	2023	FH (Fear → hope) > HF (hope → fear)	Activism intentions	Yes		FH > HF x issue relevance (high) → activism intentions
Others						
Dillard & Shen [62]	2018	Fear	Intention and behavior to ascertain one’s vaccination status	Yes		
Ecker et al. [63]	2023	Fear	Belief in the vaccine–autism link	No		
Ort & Fahr [64]	2018	Fear	Attitude toward vaccination	Yes		
Sato and Takasaki [6]	2021	Fear	Vaccine uptake (−), risk perception of disease	Yes		Fear → vaccine uptake (−) x cash incentive (low)

Note: For significance: Yes = *p* ≤ 0.05, No = not significant, NR = Main effect of fear not reported. VI = vaccination intention, PBC = Perceived Behavioral Control, * investigated both influenza and COVID-19.

**Table 3:** Main effect of fear appeal on vaccination intention.

		Yes *n*(%)	No *n*(%)	χ^2^	*p*-Value
Vaccine type	COVID-19	10(47.6)	11(52.4)	1.117	0.290
	Others	8(66.7)	4(33.3)		
Country	USA	5(33.3)	10(66.7)	6.141	0.046 *
	China	7(87.5)	1(12.5)		
	Others	4(50)	4(50)		
Country	USA	5(33.3)	10(66.7)	6.135	0.013 *
	China	7(87.5)	1(12.5)		
Culture	Western	7(35)	13(65)	6.229	0.012 *
	Non-Western	9(81.8)	2(18.2)		
Population	Student	4(66.7)	2(33.3)	0.795	0.672
	Female	2(40)	3(60)		
	General Adult	12(52.2)	11(47.8)		

* *p* < 0.05; Yes = significant at the 0.05 level; *n* = number of studies.

## Data Availability

The data generated in this study are available by contacting the first author, Yam B. Limbu, if requested reasonably.
